# Mr. Morgan, on Vaccination

**Published:** 1804-01-01

**Authors:** John Morgan

**Affiliations:** Surgeon. Ipswich


### *To the Editors of the Medical and Physical Journal.*

GENTLEMEN,

THE vast utility of the Vaccine Inoculation, its safety and efficacy, are now so established, that I should not have solicited a space in your excellent Miscellany of next month for these few lines, had either my reading or other information furnished me with a fact similar to the subject of the subsequent remarks.

On September 16, I was sent for to visit the wife of N. Obey, of Rushmere, near this town. She had then sickened five days of the natural small-pox. She nursed at her breast a strong, florid child, seven months old. The pustules on the mother being numerous, I entertained some apprehensions for the safety of her offspring, both from its habit and age. I explained instantly to the parents the great probable advantage of vaccine inoculation, founded on the hope that the infant was not yet infected with the small-pox. Parental affection was soon persuaded to listen to a voice that promised deliverance from the threatened evil, and I was permited to pursue the plan I thought advisable for the infant's safety. Having luckily in my pocket some cow-pock matter, I inoculated both arms with it. On the fourth day I had the satisfaction to observe both arms had taken the infection. On the eighth day the efflorescence was complete, and the child apparently free from indisposition, having experienced no other inconvenience than very slight fever and trifling pain in the axilla.

During the whole time the child continued to suck, and to be sensible of little suffering or uneasiness from its complaint. On the ninth day, when the mother was still ill of the small-pox, the infant was perfectly well.

